# Understanding and measuring symptoms and health status in asthma COPD overlap: content validity of the EXACT and SGRQ

**DOI:** 10.1186/s41687-018-0038-5

**Published:** 2018-04-11

**Authors:** Linda Nelsen, Adam Gater, Charlotte Panter, Chloe Tolley, Laurie Lee, Steven Pascoe

**Affiliations:** 1 Value Evidence and Outcomes, GSK, Collegeville, PA USA; 2 Patient-Centered Outcomes, Adelphi Values, Bollington, Macclesfield, Cheshire UK; 3 Research and Development, GSK, Stevenage, Hertfordshire UK; 4 Respiratory Medicines Development Center, GSK, Research Triangle Park, NC USA

**Keywords:** Asthma-COPD overlap, Chronic obstructive pulmonary disease, Impacts, Patient-reported outcome, Symptoms, Health-related quality of life

## Abstract

**Background:**

Asthma-chronic obstructive pulmonary disease overlap (ACO) differs from asthma and chronic obstructive pulmonary disease (COPD) in demographics, phenotypic characteristics and outcomes, yet the patient experience of ACO is poorly characterized. We aimed to understand and compare the patient experience of symptoms and domains of impact in ACO relative to COPD, and assess the content validity of existing patient-reported outcome (PRO) instruments in ACO.

**Methods:**

This US qualitative, interview study included patients who met American Thoracic Society/European Respiratory Society spirometric criteria for COPD. Additionally, patients with ACO demonstrated reversibility (forced expiratory volume in 1 s [FEV_1_] increase ≥ 12% and ≥ 200 mL) to albuterol/salbutamol and an FEV_1_/forced vital capacity ratio < 0.7. Patients took part in concept elicitation (CE) to explore symptoms and impacts of obstructive lung disease. The Exacerbations of Chronic Pulmonary Disease Tool (EXACT), St George’s Respiratory Questionnaire (SGRQ) and a daily wheeze assessment were cognitively debriefed to assess relevance and comprehensiveness. Interviews were analyzed using Atlas.Ti. Concept saturation was evaluated at the symptom level.

**Results:**

Twenty patients with ACO and 10 patients with COPD were recruited. Patients from both groups indicated that shortness of breath was their most frequent and bothersome symptom. The most frequently reported symptoms in both groups were shortness of breath, cough, wheezing, difficulty breathing, mucus/phlegm, chest tightness, and tiredness, weakness or fatigue. The onset, severity, frequency and duration of symptoms were consistently described across both groups, although a higher proportion of patients with ACO experienced exacerbations versus those with COPD. Impacts on daily living, physical impacts and emotional impacts were commonly described (ACO: 90–100%, COPD: 80–100%). Concept saturation was achieved in both groups. Overall, the EXACT, SGRQ and daily wheeze assessment were well understood and relevant to most patients with ACO or COPD (50–100%) and patients generally found the assessments easy to complete. The PRO instruments adequately captured symptoms described during CE, demonstrating high content validity in ACO and COPD.

**Conclusions:**

Patients with ACO and COPD experienced similar symptoms and impacts. The EXACT, SGRQ and assessment of wheeze were well understood and captured concepts relevant to patients with ACO.

**Electronic supplementary material:**

The online version of this article (10.1186/s41687-018-0038-5) contains supplementary material, which is available to authorized users.

## Background

Asthma and chronic obstructive pulmonary disease (COPD) are the most common obstructive lung diseases (OLD) among adults, and cause significant disease burden [1–5]. A subset of patients with OLD experience characteristics of both asthma and COPD (fixed airflow obstruction and partial reversibility to a bronchodilator), a condition referred to as asthma-COPD overlap (ACO) [1, 2]. The prevalence of ACO among patients with OLD ranges between 15% and 55%, depending on gender, age and the diagnosis criteria used [2]. Patients with ACO often have worse quality of life than patients with asthma or COPD alone [6], and are more frequently hospitalized than patients with COPD alone [7]. However, patients with ACO are often excluded from clinical trials as they do not meet the eligibility criteria for either asthma or COPD. As a result, their responses to standard medications for these conditions are not well characterized [2].

In addition to the lack of information regarding medication responses in ACO, patients’ disease experiences and perceptions of ACO are not well understood. Patient-reported outcomes (PRO) can add substantial value in the assessment of disease/symptomatic expression, disease impacts and treatment benefit, particularly in conditions characterized by symptoms which cannot be directly observed or are best assessed based on the patient’s direct report [8]. Although many PRO instruments are available for the assessment of symptoms and disease burden in asthma and COPD, these have not been validated for use in patients with ACO and there are no condition-specific PRO instruments available to assess the experience of ACO. The Exacerbations of Chronic Pulmonary Disease Tool (EXACT) is a patient-completed diary that assesses frequency, severity and duration of exacerbations of COPD. The diary includes domains of breathlessness, cough and sputum and chest symptoms [9–12], and is often included in clinical trials of COPD. The derivative measure, the Evaluating Respiratory Symptoms in COPD (E-RS: COPD), measures daily symptoms of COPD with a total score and scores for the domains of breathlessness, cough and sputum and chest symptoms. Other PROs commonly used to assess chronic airway diseases include the St George’s Respiratory Questionnaire (SGRQ), which assesses symptoms, activity and impacts on health status in COPD and asthma [13], and daily assessments of wheeze, which are frequently used as outcomes in asthma trials.

For a PRO instrument to be accepted for use in medical product development, its content must first be validated in the target population for which its use is intended, as per US Food and Drug Administration (FDA) PRO and International Society for Pharmacoeconomics and Outcomes Research (ISPOR) guidelines [14–17]. This is typically achieved through qualitative research studies with members of the target population, and ensures that the PRO accurately assesses the concepts that are most important to the patient population in a manner that is interpreted correctly and consistently by the patient. The aims of this study were to further understand the patient experience of symptoms and impacts associated with ACO and to assess whether existing PRO instruments used in asthma and COPD independently have adequate content validity for use in patients with ACO, such that they may be used as outcomes in future clinical trials. Lastly, the aim was to develop a draft conceptual model to reflect the disease experience of patients with ACO relative to those with COPD, and highlight any differences between the two groups.

## Methods

### Study design

This was a qualitative, non-interventional interview study (GSK ID: HO-13-9398) involving patients with ACO and COPD in the USA. Patients were scheduled to take part in a 90-min face-to-face interview to discuss their disease experience and to complete a selection of existing PRO instruments used in asthma and/or COPD. Interviews were conducted by a trained Adelphi Values interviewer.

### Recruitment

Patients were recruited between May and August 2015 via clinician referrals from four sites used for GSK’s ongoing clinical trials for asthma, ACO or COPD. Patients who had failed screening for other GSK trials on the basis of not meeting the eligibility criteria for asthma or COPD were targeted for recruitment.

### Ethics

This study was approved by an independent ethical review board in the USA (IRB number: ADE2–15-022) and was conducted in accordance with FDA PRO guidelines [17], ISPOR guidelines for development and evaluation of PRO measures [14–16] and with the ethical standards laid down in the 1964 Declaration of Helsinki and its later amendments [18]. All patients provided written informed consent prior to any collection of data.

### Patient sample

At screening, all patients were ≥ 18 years of age and met the American Thoracic Society (ATS)/European Respiratory Society (ERS) diagnostic criteria for COPD [19]. Patients with COPD alone had a pre-forced expiratory volume in 1 s (FEV_1_)/forced vital capacity (FVC) ratio of < 0.7, < 12% reversibility and < 200 mL increase in FEV_1_ to albuterol/salbutamol within 6 months prior to study start. Patients with ACO also had evidence of an asthmatic component as demonstrated by spirometry, reversibility and current therapy. Specifically, patients were required to have a post-bronchodilator morning FEV_1_ ≥ 50% and ≤ 80% of the predicted normal value [20] and a pre- and post-bronchodilator FEV_1_/FVC ratio < 0.7; reversibility defined as ≥12% and ≥ 200 mL increase in FEV_1_ to albuterol/salbutamol within 6 months prior to study start; to be receiving inhaled corticosteroid (ICS)-containing therapy for ≥12 weeks prior to screening and on either a stable dose of ICS monotherapy or a stable dose of ICS in combination with adjunctive therapy within 4 weeks prior to screening. Patients were not required to have received a diagnosis of ACO from their physician prior to screening.

Patients with current respiratory infections, and those who had experienced an exacerbation requiring oral corticosteroids or antibiotics for ≥3 days or parenteral corticosteroids in the week prior to screening or an exacerbation requiring hospitalization or an emergency department visit in the month prior to screening were excluded, as were those who had undergone lung resection within 12 months prior to screening or used oxygen therapy for ≥12 h/day.

Sampling quotas, including those related to sociodemographic characteristics, disease severity and smoking status, were employed to ensure a diverse and representative patient sample. The study aimed to recruit at least 40% females in both groups, with at least 25% of the ACO group and at least 20% of the COPD group <60 years of age, and at least 25% of the ACO group and at least 20% of the COPD group to have experienced a disease exacerbation in the 6 months prior to screening.

### Interview procedure

Interviews lasted approximately 90 min and followed a semi-structured interview guide. The first 30–40 min of the interview comprised concept elicitation (CE), which involved broad open-ended exploratory questions designed to facilitate spontaneous and non-biased elicitation of content regarding the patient experience of symptoms and impacts relating to their condition (ACO or COPD). For example, “Tell me about a typical day with ACO/COPD”. If patients mentioned a symptom or impact relevant to their disease experience, more direct and focused questions were asked to ensure that the patient’s experience was explored in depth. Interviews captured information regarding the onset, duration, location, frequency, severity, bother and impact of symptoms and their associated impact on functioning and other key domains of health status. Following this, patients were asked further questions about topics that may not have been previously discussed during the course of the interview. For example, “Have you noticed anything that triggers your symptoms?”. The last 40–50 min of the interview consisted of cognitive debriefing (CD) of the EXACT, SGRQ and daily assessment of wheeze. For each separate PRO, patients were given the standard instructions that come with the questionnaire, and asked to describe their understanding of each instruction and item, before completing each questionnaire. This was achieved using a ‘think aloud’ exercise in which they were asked to speak aloud their thoughts as they read each instruction and completed each item. Following this, patients were asked detailed debriefing questions about the definitions/meanings of terms, understanding/clarity of instructions and item wording, and the relevance of concepts captured in each of the questionnaire items. Furthermore, the appropriateness of response options and recall periods were explored. Additional questions assessed conceptual coverage, i.e. whether patients thought any items relating to symptoms or the impact of respiratory disease on their daily lives were missing on the EXACT and SGRQ. Due to the large number of items in the SGRQ, coverage was assessed using general questions about the questionnaire as a whole.

### PRO instruments

The EXACT is a 14-item patient-completed diary used to evaluate unreported and reported exacerbations of COPD [9, 10]. The 11 respiratory symptom items, referred to as the E-RS:COPD scale, can also be scored separately to evaluate symptom outcomes in clinical trials; it includes overall symptoms, as well as specific domains assessing breathlessness, cough and sputum, and chest symptoms [11, 12]. Each item in the EXACT is scored on a 5- or 6-point ordinal scale, with a total score based on all 14 items transformed to a 0–100 scale for ease of use (lower scores indicate better status) [10]. For this study, the items comprising the EXACT were evaluated for relevance to symptoms experienced by patients with ACO. The EXACT was not evaluated as a measure of exacerbations, nor was the E-RS:COPD assessed as a measure of respiratory symptoms in ACO.

The SGRQ (standard version) is a self-administered, health status questionnaire consisting of 50 items with 76 weighted responses split across three domains assessing symptoms, activity and impacts in COPD and asthma, with lower scores indicating better status [13]. The first eight questions address the frequency of respiratory symptoms over a variable preceding period and the remaining questions address the patient’s current state of disturbance to daily physical activity (activity score) and psychosocial function (impact score). A total score based on all 50 items is transformed to a 0–100 scale with lower score indicating better health status.

Daily assessment of wheeze was a single self-administered question: ‘Did you wheeze today?’ utilizing a 5-point verbal rating scale (ranging from ‘not at all’ to ‘almost constantly’), similar to response options used in the EXACT.

Permission to use the EXACT and SGRQ instruments was obtained from the copyright holders (Evidera for the EXACT tool and St George’s Hospital for the SGRQ) prior to use in this study.

### Qualitative data analysis

All interviews were digitally-recorded and transcribed verbatim to allow for qualitative analysis using Atlas.Ti software (Version 7, Scientific Software Development GmbH, Berlin, Germany) [21]. CE interviews were analyzed using thematic analysis. To ensure that all relevant concepts were captured, conceptual saturation (i.e. the point at which no substantially new concepts emerged beyond what had already been mentioned) was evaluated for the CE component of the interview at the symptom level. Successive sets of five interviews were compared for the ACO group and sets of three/four interviews were compared for the COPD group. For the analysis of CD interviews, codes were first assigned to each item/instruction followed by separate codes indicating relevance, patient understanding and any suggestions for change. Due to the small and unequal sample sizes across groups, this study was not designed to compare data quantitatively. Patient identification numbers were used to preserve patient anonymity and provide a reference for patient quotes. They were assigned based on age, sex (male [M] or female [F]) and condition (ACO or COPD). For example, F-45-COPD is a 45-year-old female patient with COPD.

The findings from the CE interviews were used to inform the development of a conceptual model to reflect the patient experience of ACO relative to COPD based on symptoms, impacts and triggers of the disease, and was used to determine any differences in the patient experience between the two populations. Furthermore, the model was used to evaluate the conceptual coverage of the EXACT and SGRQ for use in ACO.

## Results

### Patient population

A demographically and clinically diverse sample of patients was recruited, which included a total of 20 patients with ACO and 10 patients with COPD (Table 1). Patients in both groups had a similar mean age (ACO: 58.5 years, COPD: 64.4 years), were mostly male and had a variety of education levels ranging from some high school to a university or college degree. Patients with ACO experienced more (*n* = 10) exacerbations requiring corticosteroids in the last 12 months versus patients with COPD (*n* = 1). There was a higher proportion of patients with COPD who were current smokers (60%) compared with ACO (25%). The baseline lung function (FEV_1_) was similar in both groups.

**Table 1:** Patient demographics and clinical characteristics

	Patients with ACO (*n* = 20)	Patients with COPD (*n* = 10)	Total (*n* = 30)
Demographics			
Age (years), mean (range)	58.5 (45–72)	64.4 (52–79)	60.5 (45–79)
Gender, % (n)			
Male	65 (13)	60 (6)	63 (19)
Ethnicity, % (n)			
Not Hispanic/Latino	95 (19)	90 (9)	93 (28)
Race, % (n)			
African American/African	10 (2)	30 (3)	17 (5)
White – Arabic/North African	5 (1)	–	3 (1)
White – White/Caucasian/European	80 (16)	60 (6)	73 (22)
Other: White	–	10 (1)	3 (1)
No response	5 (1)	–	3 (1)
Education, % (n)^a^			
Some high school	15 (3)	20 (2)	17 (5)
High school diploma/GED	20 (4)	50 (5)	30 (9)
Some years of college	35 (7)	–	23 (7)
Certificate program	10 (2)	10 (1)	10 (3)
University/college degree (2 or 4 year)	20 (4)	20 (2)	20 (6)
Vocational training	10 (2)	–	7 (2)
Work status, % (n)^a^			
Working full time	55 (11)	10 (1)	40 (12)
Looking for work	–	10 (1)	3 (1)
Full-time homemaker	5 (1)	–	3 (1)
Not working due to respiratory condition	15 (3)	10 (1)	13 (4)
Retired	20 (4)	50 (5)	30 (9)
Other: disabled or odd jobs around neighborhood	5 (1)	20 (2)	10 (3)
General health, % (n)			
Very good	10 (2)	10 (1)	10 (3)
Good	55 (11)	50 (5)	53 (16)
Fair	30 (6)	30 (3)	30 (9)
Poor	5 (1)	10 (1)	7 (2)
MMRC dyspnea scale, % (n)^a^			
0	50 (10)	30 (3)	43 (13)
1	55 (11)	70 (7)	60 (18)
2	20 (4)	30 (3)	23 (7)
3	15 (3)	20 (2)	17 (5)
Clinical characteristics			
Primary disease, % (n)			
Asthma	45 (9)	–	30 (9)
COPD type: Chronic bronchitis	55 (11)	100 (10)	70 (21)
COPD type: Emphysema	10 (2)	50 (5)	23 (7)
First use of a maintenance therapy inhaler (years)			
Mean (range)	40.5 (14–64)	42 (8–60)	42 (8–64)
Asthma diagnosis, % (n)			
Yes	60 (12)	40 (4)	80 (16)
Mean (range) age when first diagnosed	23 (5–62)	30.5 (1–57)	25 (1–62)
COPD diagnosis, % (n)			
Yes	65 (13)	100 (10)	77 (23)
Mean (range) age when first diagnosed	49 (25–63)	52.5 (38–71)	50 (25–71)
Smoking history, % (n)			
Never smoked	25 (5)	–	16 (5)
Current smoker	25 (5)	60 (6)	37 (11)
Former smoker	50 (10)	40 (4)	47 (14)
Smoking history, pack years			
Mean (range)	35 (4–92)^b^	56 (33–90)^c^	43 (4–92)
Exacerbations that required corticosteroids			
In the last 12 months, n	10	1	11
Mean (range)	0.9 (0–4)	0.1 (0–1)	0.6 (0–4)
In the last 3 months (n)	3	0	3
Mean (range)	0.15 (0–1)	–	0.1 (0–1)
Exacerbations that required hospitalization			
In the last 12 months (n)	2	2	4
In the last 3 months (n)	0	1	1
Lung function FEV_1_ (% predicted), mean (range)	54 (1.6–68)	50 (1–79)	53 (1–79)
Reversibility (L), mean (range)			
Pre-albuterol lung function (L in FEV_1_)	1.7 (1.1–2.7)	1.7 (1.1–2.6)	1.7 (1.1–2.7)
Post-albuterol lung function (L in FEV_1_)	2.1 (1.3–3.1)	1.7 (0.8–2.5)	1.9 (0.8–3.1)
Percent change	19.2 (11.5–38.2)	0.2 (−23.1–7.8)	12.8 (−23.1–38.2)
Clinician rated severity of COPD, % (n)			
Moderate	100 (20)	70 (7)	90 (27)
Severe	–	30 (3)	10 (3)

*ACO* asthma-COPD overlap, *COPD* chronic obstructive disease, *FEV*_*1*_ forced expiratory volume in 1 s, *GED* General Educational Development, *MMRC* Modified Medical Research Council

^a^More than one option could be selected

^b^n = 15 (current and former smokers)

^c^n = 10 (current and former smokers)

### Concept elicitation

#### Terminology and understanding

Patients with ACO (*n* = 20) referred to their condition as asthma (*n* = 7), COPD (*n* = 7), exercise-induced asthma (*n* = 2) or chronic bronchitis (*n* = 1); only three patients referred to their condition using both ‘asthma’ and ‘COPD’. The majority (12/20) of patients questioned about their understanding of ACO had not heard of the condition: *“I’ve never really heard of that, you know, uh, of, of them overlapping. And I’m just not familiar with that at all”,* (patient ID: M-65-ACO)*.* Of the 10 patients with COPD, nine defined their condition as ‘COPD’ and one called it a breathing disorder: *“I just call it a breathing disorder because basically everyone that I’m around knows me,”* (patient ID: M-65-COPD).

#### Symptoms

**Fig. 1:**
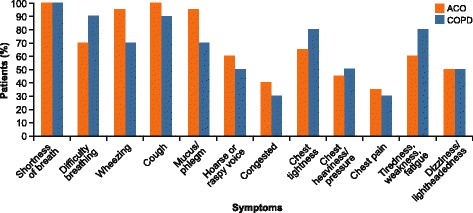
Percentage of patients with ACO and COPD reporting specific respiratory symptoms. ACO, asthma-COPD overlap; COPD, chronic obstructive pulmonary disease

The most frequently reported symptoms for patients with ACO and COPD were shortness of breath, cough, wheezing, difficulty breathing, mucus/phlegm, chest tightness, and tiredness, weakness or fatigue (Table 2; Fig. 1). Patients spontaneously reported the vast majority of these symptoms, suggesting that these are the symptoms most important to patients with ACO and COPD.

**Table 2:** Symptoms discussed during concept elicitation interviews

Sign/symptom	Patients with ACO, n (%) (*N* = 20)	Patients with COPD, n (%) (*N* = 10)
Shortness of breath	20 (100)	10 (100)
	*“My airway is restricted it seems like when I’m, when I’m breathing in. And of course, when you breathe in and you can’t take enough air in”* (M-65)	*“I’m just more or less just pulling in air. Well really I’m not even doing that but I’m just gasping for air”* (F-78)
Difficulty breathing	14 (70)	9 (90)
	*“You’re just wanting that next breath. You want everybody away from you and it’s just really it’s are you going to die, are you going to suffocate right here. Suffocation is, is a, uh, common factor. Uh, because it, it feels like everything is right on your face. It’s, it’s like mask”* (M-53)	*“I was having a hard time breathing. I had an elephant sitting on my chest and I just seemed like I couldn’t get hardly anything done and that’s when we decided I needed to have inhalers”* (M-52)
Wheezing	19 (95)	7 (70)
	*“Um, kind of like, uh, you, you take a deep breath but it kind of makes a high pitched sound going in. Like your throat’s closing or something.”* (M-47)	*“It’s just a rattling in my chest, it’s, that’s all it is.”* (M-60)
Cough	20 (100)	9 (90)
	*“It’s like you’re trying to… pull something out of your lungs, you know? Trying to—you feel like something’s obstructing it and you’re really coughing deep to try to dislodge something”* (F-48)	*“I would cough using my muscles, but sometimes it hurt when I cough* (M-58)*”*
Mucus/phlegm	19 (95)	7 (70)
	*“Well on a day like today my throat is not clear and I can, I can feel something here”* (M-62)	*“nasty little stuff, yellow and green and, you know, and some of it’s coming out of my nose, out of my throat, out of my chest”* (M-58)
Vocal Changes	12 (60)	5 (50)
	*“I can’t yell as good as I used to, you know, or, or call out to somebody. I used to have a voice that would carry for, forever. And, uh, I can’t do that anymore.”* (M-65)	*“It feels like I can’t get my voice out some. That’s what it really feels like.”* (F-53)
Congestion	8 (40)	3 (30)
	*“congestion in my lungs... feels really hard to breath, and it, it’s really uncomfortable”* (M-44)	*“[congestion] I have that in my throat. I wake up in the mornings, you know, and just feel like I’m choking sometimes”* (F-65)
Chest tightness	13 (65)	8 (80)
	*“It’s like somebody takes and just squeezes your lungs and they don’t expand so that you can get the air in”* (F-55)	*“I get a little tight, feels like something pushing on me, you know, kinda like some people will probably tell you they’re about to have a heart attack, you know, that’s just the way you feel”* (M-67)
Chest heaviness or pressure	9 (45)	5 (50)
	*“I have pressure in my chest, but where I’m coughing so much, making my chest so sore can’t hardly breathe.”* (M-59)	*“Severe symptoms is the elephants that you can’t get off your chest. It’s really tight and pressurized and you have to use your inhaler like, every hour for a while until you get things opened up.”* (M-52)
Chest pain	8 (40)	3 (30)
	*“it’s just a sharp pain that just shoots across my chest”* (M-62)	*“It gets real tight, and it hurts”* (F-53)
Tiredness, weakness or fatigue	12 (60)	8 (80)
	*“Sometimes you don’t feel like going to the bathroom, you know… you can go into bed... it’s just tiredness. I think it’s more tiredness than it’s lethargy”* (M-53)	*“I’m getting run down. I’m tired during the day, you know, and I have to sit down and take it easy… I can’t do what I want to do”* (M-58)
Dizziness or lightheadedness	10 (50)	5 (50)
	*“I have to sit down or whatever, you know, because I don’t wanna fall. But yeah, I have, uh, felt dizziness in the—like even doing my inhaler I might have to just wait a second in between now and then”* (F-57)	*“You just feel like you’re going to fall over –..And you kind of walk sideways”* (F-65)

Quotes are followed by a patient identifier. Patient identification numbers are coded as Male (M) or Female (F) followed by age. For example, F-45 is a quote from a 45-year-old female

Patients with ACO most commonly reported shortness of breath (100%), cough (100%), wheezing (95%) and mucus or phlegm (95%), whereas patients with COPD most commonly reported shortness of breath (100%), difficulty breathing (90%), cough (90%), chest tightness (80%) and tiredness, weakness or fatigue (80%). Overall, the proportion of patients with ACO and COPD reporting each of the core symptoms did not differ greatly although, interestingly, wheezing and mucus or phlegm were experienced by 19/20 patients with ACO (95%) compared with 7/10 patients with COPD (70%). However, there were no substantial differences in how patients described these symptoms in relation to onset, severity, frequency and duration. It is also important to acknowledge that, while appropriate for qualitative exploration, the small sample sizes limit ability to interpret any quantitative differences (in terms of symptom relevance) observed between groups.

Shortness of breath was described as the most frequent, most bothersome and worst symptom by the greatest number of patients in both groups. For example: *“in work, or run around recreating, you know, I’ll always get into some situation where I’ve got to stop for a second and catch my breath”* (patient ID: M-57-ACO) and *“I mean, that’s, the, probably the shortness of breath is the most irritating”* (patient ID: M-60-COPD)*.*

Overall, there was a high level of consistency in the concepts and sub-concepts reported across interviews, including for the symptom of wheezing. Patients in both the ACO and COPD groups described wheeze in terms of the sound of breathing, referring to the volume and pitch (“*you take a deep breath but it kind of makes a high-pitched sound going in. Like your throat’s closing or something”* [patient ID: M-47-ACO]), or describing it as “*a rattling in my chest*” (patient ID: M-60-COPD). Wheeze was also described by patients with ACO in terms of feeling obstruction in the airway, for example, “*It feels like somebody is giving you a bear hug and squeezing the air out of you*” (patient ID: F-48-ACO).

In the ACO group, saturation of symptoms was achieved after the third set of interviews, with no new concepts emerging in the fourth set of interviews. In the COPD group, most of the concepts and those concepts considered to be of primary clinical relevance were captured in the first set of interviews, but chest pain and runny nose emerged in the final set of interviews; this is unsurprising given the smaller sample size.

#### Bad day

In total, 12 (60%) patients with ACO and 9 (90%) patients with COPD were asked to describe the symptoms associated with a “bad day”. For the 12 patients with ACO, symptoms included shortness of breath (*n* = 6), wheezing (*n* = 4), chronic cough (*n* = 4), chest congestion or phlegm (*n* = 3) and chest heaviness (*n* = 3). In comparison, a bad day for the 9 patients with COPD mainly included difficulty breathing or shortness of breath (*n* = 6).

#### Triggers

All patients discussed factors that trigger their COPD or asthma symptoms, which included seasons or weather (ACO 95%; COPD 80%), physical activity (ACO 85%; COPD 90%) and smoke (ACO 80%; COPD 70%). Overall, there were no clear differences between the triggers reported by patients with ACO and COPD.

#### Exacerbations

Twelve (60%) patients with ACO and eight (80%) patients with COPD spontaneously mentioned or were probed to talk about exacerbations. Of these patients, 14 patients said they had experienced at least one exacerbation. There was a higher proportion of patients with ACO (11/14, 79%) who discussed experiencing an exacerbation or numerous exacerbations compared with COPD (3/14, 21%). Patients referred to an exacerbation in various ways, including being unable to breathe (*n* = 5), an asthma attack (*n* = 3), a coughing attack (*n* = 2), an attack (*n* = 2), a breathing attack (*n* = 1), an exacerbation (*n* = 1) or getting ‘sick’ (*n* = 1). Four patients referred to the duration of an attack, with three describing it as lasting for 2–3 weeks and one describing it as lasting from minutes to an hour, suggesting that there may be some inconsistency in what patients consider to be an attack or exacerbation. Six of the 20 patients who discussed exacerbations reported never experiencing an exacerbation. Of these, five were in the COPD subgroup (83%). One patient with COPD reported: *“It doesn’t, it hardly, it doesn’t ever spike. I mean, it gets pretty bad but it doesn’t spike, it doesn’t have any, you know, changes like that, I don’t think,”* (patient ID: M-67-COPD).

#### Impacts

**Fig. 2:**
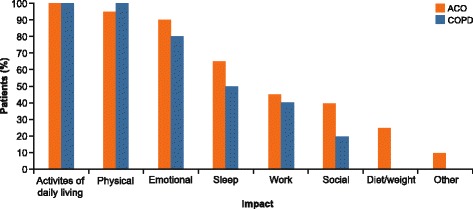
Percentage of patients with ACO and COPD reporting specific impacts. ACO, asthma-COPD overlap; COPD, chronic obstructive pulmonary disease

The symptoms of ACO and COPD impacted many aspects of daily life. Patients in the ACO group were younger and more likely to be in paid work, which may have affected the impact of respiratory disease on daily activities. The most frequently reported impacts in both groups were activities of daily living (ACO: 20/20, 100%; COPD: 10/10, 100%), physical impacts (ACO: 19/20, 95%; COPD: 10/10, 100%) and emotional impacts (ACO: 18/20, 90%; COPD: 8/10, 80%) (Fig. 2). Other domains of health status that were impacted included sleep (ACO: 13/20, 65%; COPD: 5/10, 50%), work (ACO: 9/20, 45%; COPD: 4/10, 40%) and social activities (ACO: 8/20, 40%; COPD: 2/10, 20%).

Overall, there were no major differences in the way that ACO and COPD were reported to affect patients’ lives. For activities of daily living, the most common impacts were hobbies (ACO: 9/20, 45%, COPD: 8/10, 80%), general daily life (ACO: 8/20, 40%, COPD: 7/10, 70%) and housework (ACO: 5/20, 25%, COPD: 6/10, 60%) in both groups, although greater proportions of patients with COPD reported these impacts.

The most commonly reported physical impact in both groups was feeling restricted or limited in general physical ability (ACO: 16/19, 84%; COPD: 6/10, 60%). Other impacts included ability to take part in sport (ACO: 14/19, 74%; COPD: 4/10, 40%) and mobility (ACO: 10/19, 53%; COPD: 5/10, 50%), with fewer patients with COPD reporting these impacts compared with patients with ACO. Only patients with ACO reported problems with diet and weight as a result of doing less physical exercise (5/19, 26%).

In the ACO group, the most frequently reported emotional impacts were frustration (9/18, 50%), feeling scared (8/18 44%), and embarrassment (6/18, 33%). In comparison, the COPD subgroup frequently reported feeling scared (4/8, 50%), sadness/depression (4/8, 50%) and feeling annoyed (3/8, 38%). A greater proportion of patients with ACO (9/18, 50%) reported feeling frustrated due to impacts on their daily life compared with one (13%) patient with COPD, who discussed frustration related to the financial impact of living with the condition.

A similar percentage of patients with ACO and COPD reported difficulty sleeping due to waking up in the night (ACO: 8/13, 62%; COPD: 4/5, 80%). Two patients with ACO and one patient with COPD had to wear oxygen masks during sleep.

Of those who reported impacts on work, physical limitation was the most commonly discussed factor (ACO: 5/9, 56%; COPD: 4/4, 100%). Specifically, these patients experienced shortness of breath when they over-exerted themselves and three patients with ACO took regular breaks to catch their breath. Three patients with ACO described the triggers they were exposed to at work, including fresh air (*n* = 1), chemicals (*n* = 1) and heat (*n* = 1). One of the nine patients (11%) with ACO reported having to reduce their working hours, and one of the four patients (25%) with COPD and two patients (22%) with ACO reported leaving their job due to their condition and difficulties with strenuous tasks. One patient with COPD also described difficulty in finding work due to shortness of breath.

#### Conceptual model

**Fig. 3:**
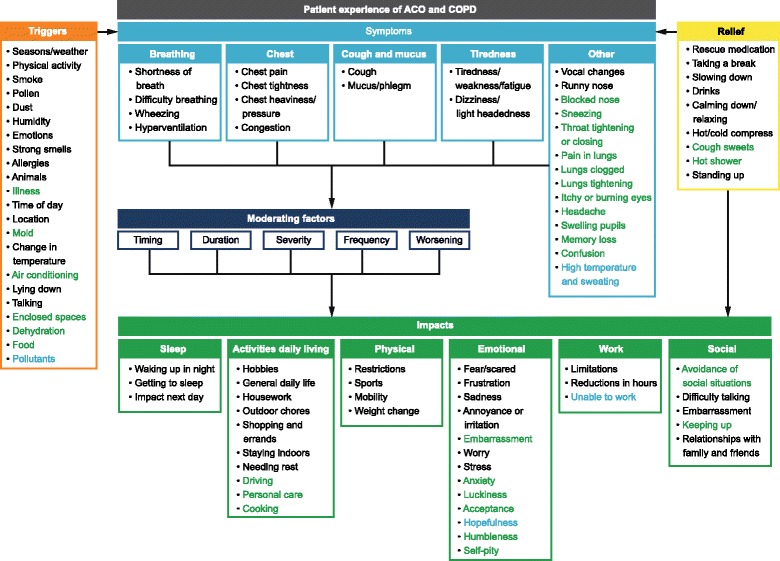
Conceptual model of patient experience and key impacts of ACO relative to COPD. Concepts highlighted in GREEN were only reported by patients with ACO and concepts highlighted in BLUE were only reported by patients with COPD; BLACK concepts was reported by both groups. ACO, asthma-COPD overlap; COPD, chronic obstructive pulmonary disease

Responses from the CE interviews informed development of a conceptual model, which provided a comprehensive picture of the patient experience of symptoms and impacts of ACO relative to COPD. The model revealed concepts that were only reported by the ACO or COPD groups (Fig. 3).

### Cognitive debriefing

The PRO measures were generally well understood and relevant to patients with ACO and COPD, with no distinguishable differences between patient groups in relevance or interpretation of items (Additional file 1: Table S1).

#### EXACT

The instructions and items of the EXACT questionnaire were well understood by both groups, and all patients generally interpreted and described all item concepts in a consistent way. Only a few minor issues were identified, including potential item redundancy, where nine patients (ACO: *n* = 7, COPD: *n* = 2) considered chest discomfort (item 5) and chest tightness (item 6) as the same concept, and three patients (ACO: *n* = 2, COPD: *n* = 1) found the term ‘discomfort’ ambiguous: *“I never quite understand what they mean by discomfort if that—if they’re meaning the pressure.... I don’t know a better way to put that, so, like I said, it maybe be pressure... but, um, discomfort, I don’t know if that means just feeling like you have to cough something up,”* (patient ID: F-48-ACO). All items were considered relevant to most patients in both groups. Item 7 (‘Were you breathless today?’) was considered relevant by the majority of patients (ACO: 16/20, COPD: 9/10), whereas Item 13 (‘Last night, was your sleep disturbed?’) was considered relevant by the least number of patients (ACO: 10/20, COPD: 5/10). Overall, patients in both groups understood the response options and generally kept to the recall period of ‘today’. While most patients (*n* = 12) said they were thinking about ‘today’ when they chose their answers, others reported the last 24 h (*n* = 2), yesterday (*n* = 1), the last couple of months (*n* = 1), the last week (*n* = 1), ‘how I feel mostly’ (*n* = 1) and since the symptoms began (*n* = 1). In addition, one patient stated that the recall period was dependent on when the diary was completed. Most of the patients who provided feedback thought the diary was quick and easy to complete (18/19), and most patients (15/25) stated that they thought no concepts were missing. The remaining 10 patients (ACO: *n* = 9, COPD: *n* = 1) suggested that concepts were missing, however none were suggested more than once, with the exception of wheezing (*n* = 2), and dizziness (*n* = 2). Missing concepts reported by individual patients with ACO included sinus problems, hoarse or raspy voice, feeling disoriented, throat clearing or irritation, animal hair as an asthma trigger, the need to use rescue medication before the day has begun, changes in asthma medication use, and response time for recovery following asthma medication.

#### Daily assessment of wheeze

The daily assessment of wheeze was understood by all patients with ACO and COPD, although three patients with ACO reported they could not accurately remember whether they had wheezed that day. Most patients thought the assessment was relevant (ACO: 17/20; COPD: 9/10); the remaining patients (ACO: 3/20, COPD: 1/10) either felt that the assessment was not relevant or did not comment. The response options were understood by all but one of the patients who commented on them (11/12). The remaining patient with COPD felt that the response options were not appropriate for the question, and suggested a dichotomous yes/no should be used. All (3/3) patients who commented on the recall period reported that they understood the recall period of ‘today’. The majority (21/22) of the patients questioned, found the assessment easy to complete.

#### SGRQ

For the SGRQ, patients generally understood the questionnaire, with some patients describing difficulty with the terminology “wheezing attacks” (3/30) and questioning the duration of an attack (2/30). However, this did not prevent these patients from completing the item based on their interpretation. All 17 patients asked considered all items relevant; three patients suggested adding ‘sometimes’ to the response options. The interpretation of the recall period was varied between patients, as some items did not include a recall period or when they did, they used “these days”. The majority (21/23) of patients asked, thought that the length of the questionnaire was reasonable. Of the 22 patients who were asked if concepts were missing from the SGRQ, 16 patients thought that no concepts were missing (ACO: *n* = 10, COPD: *n* = 6). Six patients thought that concepts were missing (ACO: *n* = 4, COPD: *n* = 2); concepts included activities/location at the onset of symptoms (ACO), getting tired when it is cold (COPD), more about cough and phlegm (COPD), impact on vacations (ACO), chest tightness (ACO) and the need to use rescue medication before the day has begun (ACO).

### Conceptual coverage of the instruments

The EXACT, SGRQ, and the daily assessment of wheeze provided good conceptual coverage of the most commonly reported symptoms in the CE portion of the interviews. During the cognitive debriefing interviews, 15 (ACO: *n* = 7, COPD: *n* = 8) and 16 (ACO: *n* = 10, COPD: *n* = 6) patients stated that no concepts were missing from the EXACT and SGRQ instruments, respectively. While vocal changes and dizziness or light-headedness were not assessed by any of these instruments, these are symptoms that are reported equally by the ACO and COPD groups. Furthermore, while these symptoms are reported by patients with ACO and COPD, these are not considered to be direct symptoms of the diseases [22].

## Discussion

According to the Global Initiative for Asthma (GINA) and Global Initiative for Chronic Obstructive Lung Disease (GOLD) 2017 guidelines, ACO is not a single disease, but represents a heterogeneous population of patients with various airway phenotypes due to a variety of different underlying mechanisms [1, 2]. To improve our understanding of ACO, the GINA guidelines are promoting research of outcomes in this patient population. This study contributes to the field by improving understanding of the patient experience of ACO and validating existing PRO instruments previously validated for asthma and COPD for use in an ACO population.

Overall, our findings show that the burden of disease is substantial in patients with ACO. Overall, there were similarities between patients with ACO and COPD in the most commonly reported symptoms (Fig. 1). Both groups commonly reported shortness of breath, difficulty breathing, wheezing, cough, mucus or phlegm, vocal changes, congestion, chest tightness, chest heaviness or pressure, chest pain and tiredness, weakness or fatigue. However, while differences between groups must be interpreted with caution given the small sample size, more patients with ACO reported wheezing and mucus or phlegm than patients with COPD (95% vs 70%), whereas more patients with COPD reported difficulty breathing, chest tightness, chest heaviness/pressure and tiredness, weakness and fatigue than patients with ACO (80–90% vs 60–70%). This study also suggested that patients with ACO were more likely to experience an exacerbation or have a history of exacerbations than patients with COPD alone. This finding is supported by previous reports showing exacerbations are more frequent in patients with ACO than COPD [23–25]. For the impact of symptoms on patients’ daily lives, there were no substantial differences in the domains of impact experienced by patients with ACO or COPD (Fig. 2). However, patients with ACO reported problems with weight gain due to their impaired ability to take part in physical activity, whereas patients with COPD did not.

An interesting finding in our study was how few patients with ACO (*n* = 3/20) were aware that they had the condition. This could reflect the lack of an accepted definition and low awareness in the medical community [24, 26].

Overall, the PRO instruments assessed were understood by and relevant to patients with ACO. Importantly, concepts covered by these tools were consistent with the majority of symptoms reported by both ACO and COPD patient groups during CE interviews, providing novel support for their use in an ACO population. Overall, all items of the EXACT were generally well interpreted by patients with ACO, who described the concepts in a similar way to patients with COPD. The symptoms of breathlessness, cough and sputum and chest symptoms captured by the EXACT in the COPD population are consistent with previous COPD populations [10]. The use of EXACT for exacerbations and E-RS for respiratory symptoms in patients with COPD is further supported by FDA and European Medicines Agency Guidance, which qualify these PRO measures for use as exploratory endpoints in clinical trials [27, 28]. However, there was some conceptual overlap between the items of chest discomfort and chest tightness in a minority of patients (ACO: *n* = 7/20, COPD: *n* = 2/10) and three patients poorly understood ‘discomfort’ (ACO: *n* = 2/16, COPD: *n* = 1/9). Further quantitative research is therefore required to determine whether these concepts could be combined, or if the item of chest discomfort could be removed in studies of symptom severity in patients with ACO.

The daily assessment of wheeze demonstrated high content validity in patients with ACO. However, there were a small number of patients who could not remember whether they had ‘wheezed’ that day, although it is arguable that patients are likely to be more mindful of this in a clinical setting.

For the SGRQ [13], there was some variability in how patients interpreted the recall period and ambiguity about the term ‘wheezing attack’. In the CE portion of the study, patients with ACO described wheeze in terms of the quality and sound of their breathing (e.g. high-pitched, rattling or obstructive), but it will be important to establish that patients have a consistent understanding of the term ‘wheeze’ when using the SGRQ and daily wheeze assessment in clinical trials among patients with ACO. In all other respects, patients generally found the SGRQ easy to respond to and there is considerable evidence of the use of the SGRQ in respiratory research [13]. Given that patients with ACO in our study identified items missing from both the EXACT and the SGRQ, consideration should also be given to the development of an ACO-specific instrument to assess symptom burden and impact in this patient group.

It is important to note that quantitative analysis of data between the ACO and COPD groups was not the purpose of this qualitative study, and so any numerical comparisons between the two groups should be interpreted with caution as the sample sizes were small. Rather, the COPD group was included in this study to provide context for the findings in patients with ACO, since the instruments assessed in the study are established in patients with COPD. In addition, the number of patients in the ACO and COPD patient groups was not equal, and, as expected, the two groups had different clinical profiles, further limiting any comparison between groups. For example, patients with COPD had more severe obstruction and included more current or former smokers. It may have been expected that the patients with COPD would therefore have been more likely to experience shortness of breath, cough, sputum and chest congestion than patients with ACO in this study, but in fact the reverse was true. Patients with COPD were also older and fewer were in employment compared with the ACO group, suggesting that the impact of the disease could be different between groups. The study included three further limitations. Firstly, definitions of ACO are not standardized, so we developed inclusion criteria that would allow us to identify patients with ACO from among a larger cohort of patients with COPD; our findings therefore cannot be generalized to populations identified using different criteria to define ACO. Secondly, patients with asthma alone were not included in the study, which meant that direct comparisons with this group could not be made, although the SGRQ has been previously validated in patients with asthma [13, 29]. Thirdly, the symptoms and impacts in the conceptual model were broad in both populations, with patients experiencing a range of breathing and chest symptoms, as well as tiredness/fatigue. This may lead to variability in PRO instrument domain scores. Despite these limitations, concept saturation was achieved during the CE interviews, and patients generally described concepts in a consistent manner, suggesting that comprehensive coverage of the symptoms and impacts of ACO was achieved with this patient sample.

## Conclusions

This is the first study to capture the patient experience of ACO using CE interviews. Our findings demonstrate that there were no substantial differences in the symptoms or domains of impact experienced by patients with ACO or COPD alone. The EXACT, SGRQ and the daily assessment of wheeze, which are existing PROs developed for COPD or asthma, demonstrated high understanding and relevance among both study populations. All minor issues that were identified with the instruments were specific to the instrument as a whole and not specific to the disease population. This study shows that the items comprising the EXACT, SGRQ and the daily assessment of wheeze have sufficient content validity and may be appropriate for the evaluation of symptoms or health status among patients with ACO. Further quantitative analysis of the properties of these PROs in an ACO population are warranted to support their use as endpoints in clinical trials investigating ACO.

## Additional file

Additional file 1: **Table S1.** Summary of responses from cognitive debriefing interviews. (DOC 92 kb)

## Abbreviations

ACO: Asthma-COPD overlap
ATS: American Thoracic Society
CD: Cognitive debriefing
CE: Concept elicitation
COPD: Chronic obstructive pulmonary disease
ERS: European Respiratory Society
EXACT: The Exacerbations of Chronic Pulmonary Disease Tool
FEV_1_: Forced expiratory volume in 1 second
FVC: Forced vital capacity
ICS: Inhaled corticosteroids
IRB: Independent Review Board
ISPOR: and International Society for Pharmacoeconomics and Outcomes Research
OLD: Obstructive lung disease
PRO: Patient-reported outcome
SGRQ: St George’s Respiratory Questionnaire
