# Supplementary material for: Understanding and measuring symptoms and health status in asthma COPD overlap: content validity of the EXACT and SGRQ
Source: J Patient Rep Outcomes. 2018 Apr 11;2:18. doi: 10.1186/s41687-018-0038-5 (PMC5935047; doi:10.1186/s41687-018-0038-5)
Supplement: Supplementary file 1 — Table S1. Summary of responses from cognitive debriefing interviews. (DOC 92 kb) [file 41687_2018_38_MOESM1_ESM.doc]

# Additional file 1

Table S1. Summary of responses from cognitive debriefing interviews

|  | **Understood item, n (%)** | | **Thought item was relevant, n (%)** | | **Understood response options, n (%)** | |
| --- | --- | --- | --- | --- | --- | --- |
|  | **Patients with ACO**  **(N=20)** | **Patients with  COPD**  **(N=10)** | **Patients with  ACO**  **(N=20)** | **Patients with COPD**  **(N=10)** | **Patients with  ACO**  **(N=20)** | **Patients with  COPD**  **(N=10)** |
| **EXACT** |  |  |  |  |  |  |
| **Item 1: Congestion** | 20 (100) | 10 (100) | 14 (70) | 7 (70) | 17 (85) | 9 (90) |
|  | *“So, for me, did your chest feel congested today? That would be if I was feeling like I needed to cough and kind of bring something up. So, for today, I would say not at all because I have not had to cough today at all”* (F-48) | *“It means your chest feeling tight and rattling, and, you*  *know, full mucus”* (M-64) | *“When I got up this morning I was congested.*  *Uh, until I had my treatment and got to coughing and getting a lot of that out of my chest”* (F-61) | *“once in a while it feels congested, like I say, if I'm in dust a lot or dusty stuff, dusty and pollen”* (M-52) | *“moderately is what I usually have when I'm getting a cold, I'm starting to get a cold. Severely is when I've got that cold, and extreme is you need to go to the doctor because it's so bad you can't hardly breathe, you know, and you can't clear it up at all, you just cough”* (F-63) | *“Moderately would be that I was coughing up stuff, but it would clear up my lungs, so I could breathe easier. That would be moderate. Severe would be where you, where I cough up phlegm and black stuff or whatever and I still can't breathe”* (M-52) |
| **Item 2: Frequency of cough** | 20 (100) | 10 (100) | 16 (80) | 7 (70) | 18 (90) | 9 (90) |
|  | *“It’s just when I wake up that first 30 minutes out of that first hour or something that I get up...And that’s when I be coughing and I cough. I get up and I be coughing about an hour or so”* (M-72) | *“You know, I don’t remember coughing today. I might have but I can’t remember, so I’d have to say not at all, I guess”* (M-67) | *“my answer would be frequently... Cause I cough all the time”* (F-46) | *“It's back to the dusty, dirty, what I did the day before stuff, and then it would be in the morning like when I first get up”* (M-52) | *“And rarely would be like once or twice throughout the entire day. Occasionally would be a few times during the day but not a whole lot. Frequently would be like every hour or at least an almost constantly would be almost the entire day”* (F-55) | *“rarely would be once in a while, and occasionally that would be a little bit more often,*  *frequently would be I would thing every day, and almost constantly that’s just cough, cough, cough, which I never do”* (F-65) |
| **Item 3: Quantity of mucus (phlegm) expectorated** | 19 (95) | 10 (100) | 10 (50) | 6 (60) | 18 (95) | 9 (90) |
|  | *“in your throat and your chest and whatever you cough up”* (M-64) | *“Mucus to me is like the black stuff and phlegm to me is like the yellow, gross, sticky stuff”* (M-52) | *“I would put a little because this morning I did,*  *though it’s usually just in the morning when I wake up and just a little bit”* (M-47) | *“A little this morning. Like I said, this morning when I got up that's when it's—that's when I have it, in the mornings”* (M-58) | *“[a very great deal] that means I'd be coughing a lot, a lot of the stuff up. A little, that means I'm not coughing none at all hardly… Not at all, that'd be I wouldn’t be bringing up nothing”* (M-59) | *“A little would probably be half a tablespoon, um, probably some would be a tablespoon, and then a great deal would be two, two tablespoons. [What about a very great deal?] That's a half a cup right there”* (M-60) |
| **Item 4: Difficulty expectorating mucus** | 20 (100) | 10 (100) | 14 (70) | 5 (50) | 15 (75) | 10 (100) |
|  | *“that means did you have to cough a lot or clear your throat a lot to be able to actually bring it up”* (F-58) | *“Uh, it feels like a pulling feeling in your lungs, but it won't turn loose sort of say it just kind of like comes, wants to come away from whatever it's doing in there, but it won't, just won't come out”* (M-52) | *“It’s, umm, to me it’s always extremely difficult. I mean I would say, well, at least quite a bit”* (F-57) | *“If I got a cold, uh, like a chest cold or*  *whatever, pretty frequent”* (M-52) | *“Not at all, you wouldn’t have an issue every time you want to bring it up, it comes up. You know how guys do “a-phew…” Slightly, umm, that would be you don’t have that much issue to me, getting it up, I mean if you wanted to you could, if you don’t want to you wouldn’t.*  *Moderately, I would just say that it only comes up when it needs to”* (F-57) | *“Slightly you would have a little bit of trouble where you felt like you needed to but it didn’t come up. Moderately every few times you cough. I would classify quite a bit and extremely together would be virtually about every time you cough”* (M-65) |
| **Item 5: Presence of chest discomfort** | 16 (80) | 9 (90) | 14 (70) | 7 (70) | 12 (60) | 7 (70) |
|  | *“I would put slight because I already mentioned that, that I was having a bit of discomfort... The pain in here that I mentioned”* (M-65) | *“Moderate…Because it hasn't, it's been the slight, you know, congestion, but it hasn't been the, you know, full blown tightness in your chest. It's slight discomfort, you know”* (M-64) | *“I would say well it would probably be slight or moderate, you know? Just because of that. Just because I do have it. But it’s not going to end my world”* (M-57) | *“It hasn't, it's been the slight, you know, congestion, but it hasn't been the, you know, full blown tightness in your chest. It's slight discomfort, you know”* (M-64) | *“Not at all means none, slight means you’re aware of it, moderate means you can feel it, severe means um, there’s a band around you, and extreme means sort of like a heart attack”* (M-53) | *“Moderate, uh, like I say, moderate is something you're going to experience time and time during the 24-hour period. And severe, I mean, that's when you have to take the, some out to rest”* (M-64) |
| **Item 6: Presence of chest tightness** | 18 (90) | 10 (100) | 16 (80) | 5 (50) | 10 (50) | 8 (80) |
|  | *“Did your chest feel tight today? Not at all, slightly, moderately, severely, or extremely? Me, not at all. 'Cause I don’t feel any tightness”* (F-46) | *“Did your chest feel tight today? Well, I'm going to put slightly because this morning it was tight, until I used my inhaler”* (M-52) | *“Well slightly simply because when I leave here I will go out into the front room and sit for about 15 or 20 minutes before I get in my care just to try and, you know, not calm down but just, uh, regenerate, rejuvenate”* (M-65) | *“Well, I'm going to put slightly because this morning it was tight, until I used my inhaler”* (M-52) | *“The severe and the extreme would be a lot of tightness, uh, almost to the point where you would need extra help besides your medication”* (F-61) | *“Severely tight... somebody sitting on your chest, you can't get them off…you can't seem to breathe...Extremely, severe, extremely would be where I'd get really light-headed, and it, it's like a, kind of a pain…Slightly tight would be like this morning where I just, just couldn't get*  *air in the way I wanted to”* (M-52) |
| **Item 7: Presence of breathlessness** | 18 (90) | 10 (100) | 16 (80) | 9 (90) | 10 (50) | 5 (50) |
|  | *“Were you breathless today? Um, I’m going to say slightly because I was off today so, I didn’t have the stress and stuff, but I was a little breathless”* (M-47) | *“Were you breathless today? Slightly, like I said again, this morning, that's because I mowed a bunch of dusty stuff right before I went to bed”* (M-52) | *Were you breathless today? Not at all, I would say slightly on that one asking me was I breathless today”* (M-72) | *“Slightly, like I said again, this morning, that's because I mowed a bunch of dusty stuff right before I went to bed”* (M-52) | *“Slightly is what I did today. Moderately, uh, is if I am just overexerting myself, you know, and doing more than I normally do, which isn't much, and, uh, severe, uh, moderately, yeah. Severely is when I really overdo, uh, like outside”* (F-63) | *Slightly, like I said again, this morning, that's because I mowed a bunch of dusty stuff right before I went to bed.”* (M-52) |
| **Item 8: Severity of breathlessness** | 17 (85) | 10 (100) | 16 (80) | 8 (80) | 9 (45) | 6 (60) |
|  | *“Uh, on slight activities because, uh, just doing activities... That include washing, dressing, light activities, that include all of that”* (M-72) | *“I was more breathless doing strenuous activity than just sitting down or resting or anything like that”* (M-64) | *“I’d have to say breathless when resting because you just, um, active through the day that you really don’t notice it unless it actually stops you from doing whatever you’re doing”* (F‑55) | *“Well, I usually feel all right when I’m resting. Well, I might feel tight but I don’t get breathless much. Washing or dressing, yeah, maybe a little”* (M‑67) | *I guess our speaking would be light activity. So I haven't done anything strenuous yet today except get up... I'm always aware of breathlessness”* (M‑65) | *“I know during strenuous activity, I was unloading some tires, and that was strenuous activity, and I was breathless. I was breathless doing light activity, you know, getting in and out of the truck”* (M‑64) |
| **Item 9: Breathlessness during usual personal care activities like washing and dressing** | 20 (100) | 10 (100) | 12 (60) | 5 (50) | 8 (40) | 6 (60) |
|  | *“Um, so for me, not at all. Usually, you know, getting up, taking a shower, doing the dishes, vacuuming, all of those things just—they don’t affect me to be short of breath at all”* (M‑57) | *“Well, I’d say moderately. I don’t, when I’m taking a shower, I’m not really moving that fast so I don’t, you know, so I just get a little bit”* (M‑67) | *“There's days when extremely would be like, uh, mopping the floor. That really gets hard to do at times. Uh, you know, sometimes even as much as making my bed up exerts me”* (F‑61) | *“I'd have to put slightly. I mean, like I said, I was not breathless breathless, but, um, needed to breathe better”* (F‑53) | *“Moderately would be not being able to do—well struggling to get my bath and struggling to get dressed. Severely? Not being able to...Extremely would be same as severely to me”* (F‑55) | *“Slightly is when you're going to be aware of being winded, and moderately you're going to be definitely aware. Severely, you're going to stop between each step, you know, and extremely, you're too breathless to do them”* (M‑64) |
| **Item 10: Breathlessness during usual indoor activities like cleaning or household work** | 17 (85) | 10 (100) | 14 (70) | 8 (80) | 5 (25) | 3 (30) |
|  | *“Housework, no, I didn’t experience any shortness of breath when I was doing that this morning”* (M‑65) | *“Yeah, I was, I noticed it when I went and got my coffee and stuff this morning. If I put slightly, that's what I put. Uh, I could just feel that it was, I was having a little problem... just a slight tightness in my chest”* (M‑52) | *“If there was a lot of sweeping and mopping and, um, vacuuming, I’ll continuous, you know, like for two hours or something. Then there might be a chance that it might be slightly to moderately”* (F‑48) | *“Sometimes I do when I get up because I'm bad about moving a chair or the couch to clean out from under it or, or sometimes if I pick up my end table it's heavy”* (F‑78) | *“I would think somebody would put extremely if they can just barely get the bed made, get a shower, you know, put their makeup on, something like that”* (F‑48) | *“Moderately would be where I'm having problems and needed to get to my inhaler as soon as possible. Severely, severely would be one of the mornings where you can't do nothing to make it go away for a while. Uh, you have to use your inhaler and wait a while and see what*  *happens, and probably use it again”* (M‑52) |
| **Item 11: Breathlessness during activities outside the home such as yard work or errands** | 16 (80) | 10 (100) | 14 (75) | 9 (90) | 10 (50) | 5 (50) |
|  | *Short of breath today when performing your usual activities. No because I didn’t use my medicine”* (F‑55) | *“Were you short of breath today? Yes. It was never severely today, more moderate than anything”* (M‑64) | *“Moderate, you know, kind of—if I'm outside doing my work if I get short-winded or something I've got to sit down”* (M‑59) | *“Well, I'm going to say slightly because I was until I went and took care of the chickens and stuff, and I was still slightly then”* (M‑52) | *“Moderate, you know, kind of—if I'm outside doing my work if I get short-winded or something I've got to sit down”* (M‑59) | *“A lot of people have trouble with yard work or doing errands and stuff, so I think it's good*  *answers”* (M‑65) |
| **Item 12: Presence of weakness or tiredness** | 17 (85) | 8 (80) | 14 (75) | 9 (90) | 4 (20) | 3 (30) |
|  | *“Tired yes slight—I’d say slightly tired. Because I didn’t rest good last night”* (F‑55) | *“Not at all. I had a good night's sleep pretty much”* (M‑52) | *“In that, in that sense of prolonged activity, something like that, yeah I can see by then end of the day I’m probably more worn out than*  *others”* (M‑57) | *“Well I’m always weak. I gotta say severely, I guess”* (M‑67) | *“So it's slightly, as a slight discomfort. But it's not to the point that it tires me out or makes me weak”* (M‑62) | *“I would say most days, most days slightly, some days moderate, um, I don't think, I don't think I've ever felt extremely tired or weak, because, if I do, I just go to sleep.”* (M‑60) |
| **Item 13: Sleep disturbance** | 20 (100) | 10 (100) | 10 (50) | 5 (50) | 9 (45) | 5 (50) |
|  | *“Did up wake up coughing, sneezing, out of breath, can’t – can’t sleep, had to get up, set up, can’t sleep”* (M‑72) | *“My sleep is disturbed a lot but it’s more from the sleep apnea than it is from the, than the COPD”* (M‑67) | *“I would say slightly...Because it—um, actually I remember using an inhaler one time last night. Rest time was just you know anxious I think”* (F‑55) | *“Actually I wasn't sleeping good at night 'cause I couldn’t breathe at night”* (M‑58) | *“If I do get a chest cold, and I'm coughing. I'm up, but that's the cold. It's not the asthma, but, even though it may incite a wheezing, um, with the coughing and all that stuff, but it's more, uh, cold-related”* (M‑55) | *“If I had put moderately it would have meant that I had been woken up because I couldn't breathe or woke myself up from breathing…Extremely would be where I had like an attack, I call them attacks, where I couldn't breathe, uh, and I'd have to get up and go use my inhaler, uh, and sit and wait for a little bit to see if it gets better”* (M‑52) |
| **Item 14: Fear or anxiety related to respiratory condition** | 20 (100) | 10 (100) | 13 (65) | 7 (70) | 7 (35) | 2 (20) |
|  | *“I'm going to put slightly because it's on my mind, like I said, but I don't really sit and worry about it”* (F‑63) | *“I mean I don’t feel like—like I said, I know I have a problem but it's not as bad as a lot of people. And I just don’t sit around and worry about what's gonna*  *happen tomorrow”* (F‑78) | *“Not just today but all the time… As the disease itself, it worries me, but not as much as the ones I love, on what affect it has on them”* (F‑61) | *“It's not getting no worse, but it ain't get no better. So on a daily basis it's, it's got me worried”* (M‑58) | *“But I'm going to put slightly because it's on my mind, like I said, but I don't really sit and worry about it”* (F‑63) | *“if it was severe, if it was extreme, I'd probably be somewhere with an oxygen tank on or something... slightly would be there's a, you know, I mean it's always on your mind, but…you’re not really concerned about it, and not at all would be just that... you're breathing through, through your nose and mouth and everything, and everything functions as according”* (M‑64) |
| **Daily assessment of wheeze** | n=18 | n=10 | n=18 | n=10 | n=18 | n=10 |
| **Daily assessment of wheeze** | 18 (100) | 10 (100) | 17 (94) | 9 (90) | 6 (33) | 5 (50) |
|  | *“Because I can hear it wheezing and I don’t know if others can but I can and it gets irritating”* (F‑55) | *“Do, did you wheeze today? I'm probably wheezing right now. I don't know if anybody can hear it, but occasionally, I'll just say right there”* (M‑64) | *“some mornings you’re hurrying or have to go out to the garden or something so there might be a slight wheeze”* (F‑48) | *“I've wheezed today, but it's not—I was wheezing. I could feel—I mean I could hear it”* (M‑58) | *“Umm, do you—so you said frequently, so that means you experience it; how often do you experience wheezing? Probably at least, umm, every two months”* (F‑57) | *“I don’t think there's an answer to that one. I mean did I wheeze today? Yeah, but it was—you'd almost have to just write yes... According to these questions. To me that would be a yes or no question”* (F‑78) |

Quotes are followed by a patient identifier. Patient identification numbers are coded as Male (M) or Female (F) followed by age. For example, F-45 is a quote from a 45-year-old female.
